# Functional Mobility Outcomes in Telehealth and In-Person Assessments for Wheeled Mobility Devices

**DOI:** 10.5195/ijt.2020.6335

**Published:** 2020-12-08

**Authors:** Mitchell Bell, Richard M. Schein, Joseph Straatmann, Brad E. Dicianno, Mark R. Schmeler

**Affiliations:** 1 Department of Rehabilitation Science and Technology, School of Health and Rehabilitation Sciences, University of Pittsburgh, Pittsburgh, Pennsylvania, USA; 2 Human Engineering Research Laboratories, VA Pittsburgh Healthcare System, Pittsburgh, Pennsylvania, USA; 3 Department of Physical Medicine and Rehabilitation, University of Pittsburgh School of Medicine, Pittsburgh, Pennsylvania, USA

**Keywords:** Assessment, Evaluation, Functional mobility, Telehealth, Wheeled mobility device

## Abstract

The purpose of this study was to compare telehealth and in-person service delivery models for wheeled mobility devices in terms of functional outcomes. We hypothesized that clinically significant improvements in functional mobility measured by the Functional Mobility Assessment (FMA) will occur in individuals receiving both telehealth and in-person clinic evaluations. A total of 27 Veterans receiving telehealth visits were compared to 27 individuals seen in clinic, selected from a database, matching for age, gender, and primary diagnosis. All mean individual item and total FMA scores in both groups increased from Time 1 to Time 2. Within the telehealth group, all changes in individual item and total FMA scores were statistically significant, with changes in 8 of 10 items meeting threshold for clinical significance (change >1.85 points). Within the clinic group, changes in 7 of 10 individual items and total FMA scores were statistically significant, and these same 7 items met threshold for clinical significance. Change scores for individual item and total FMA scores did not differ significantly between the two groups. A larger and clinically significant change in transfer score was seen in the telehealth group, suggesting telehealth visits may confer an advantage in being able to assess and address transfer issues in the home.

In the United States, 1 in 4 adults or 61 million Americans have a disability that impacts major life activities. Specifically, about 6.8% of Americans classify their disability type as “ambulatory” (Erickson et al., 2019). With age, disability becomes more common, affecting about 2 in 5 adults age 65 and older (Okoro et al., 2018). In those aged 65 years and older, 40% reported at least one disability, and two-thirds have a mobility limitation (He et al., 2014). Older adults with mobility limitations have higher morbidity and mortality, a lower quality of life, and isolation from the world and social circles (Gill et al., 2006). Mobility limitations are also associated with lower social engagement, including using the phone and internet, visiting friends, and participating in recreational activities (Rosso, et al., 2013). Telehealth services are increasing in many areas of healthcare. Telehealth is often used to serve parts of the community that do not normally have access to medical services. These areas may have little medical support and are often far away from specialized centers. This can result in long travel times for the client due to distance, geography, and transportation options (Ekeland et al., 2010). Hatzakis et al. (2003) surveyed Veterans diagnosed with multiple sclerosis and 20% reported barrier issues with parking, distance and transportation that interfered with them receiving treatment. Furthermore, for individuals with sensation issues, prolonged sitting during travel carried the potential risk of worsening a pressure injury of the skin (Sabharwal et al., 2001). In addition, individuals with mobility impairments, such as cerebral palsy and rheumatoid arthritis reported that healthcare barriers included access to the physical environment as well to specialists to receive care (Cooper et al., 1996; Hoenig et al., 2005; O'Day et al., 2002). For these reasons, individuals delayed or avoided required treatment. Mobility restrictions and problems with accessibility were found to decrease the quality of healthcare for individuals located in rural areas (Hatzakis et al., 2003).

The Department of Veterans Affairs' (VA) runs the largest healthcare system in the United States, with over 9 million Veterans enrolled. Veterans receive care at 172 medical centers and over 1,000 outpatient clinics (U.S. Department of Veterans Affairs, 2016). In 2016, the VA had over 700,000 Veterans using a form of telehealth, 45% of whom were living in rural communities and determined to have limited access to VA healthcare. Over 900 locations and 50 medical specialties use telehealth services. Veterans have reported 88%-94% satisfaction rates with the telehealth services (U.S. Department of Veterans Affairs, 2016). With a high population of Veterans who live in rural areas, the VA has been a leader in developing, testing, and implementing new telehealth services to ensure all Veterans are receiving a high quality of care.

For telehealth to move forward, results must yield equivalent clinical outcomes to conventional in-person care to ensure that telehealth does not deliver inferior care. Meaningful control and comparison groups must be used, and patients with varying demographics (e.g., gender, age, ethnicity, race) must be included in the samples to improve the generalizability of the findings. Graham et al. (2019) provides a scoping review analyzing research on the effects and processes of telehealth wheelchair and seating assessment and the perceptions of wheelchair users and healthcare providers of telehealth. Initial studies suggest that tele-wheelchair assessment maybe as effective as in-person assessment in reaching decisions about wheelchair and seating modifications and prescriptions (Cooper et al., 2002; Malagodi et al., 1998). Cooper et al. (2002) reported no significant differences in the wheelchair and seating equipment prescribed by assessors when using remote versus in-person assessment; however, limited information about wheelchair users' health condition or complexity was provided. In a descriptive case analysis of prescribed adaptive equipment, Malagodi et al. (1998) concluded that differences between in-person and tele-wheelchair assessment were clinically insignificant based on a descriptive comparison of prescribed wheelchair/modifications. Recent studies such as Dallolio et al. (2008) used telehealth and standard care after patients were discharged from an inpatient spinal cord injury rehabilitation unit and noted some improvements in grooming, dressing and transfers for those receiving telehealth services at one site. Lastly, Schein et al. (2010) evaluated the equivalency of wheeled mobility and seating assessments delivered under two conditions: in-person at a local clinic and via telerehabilitation at a remotely located clinic. He concluded that there was no difference in wheelchair user perceived function between tele and in-person wheelchair assessment measured by the Functioning Everyday with a Wheelchair (FEW) outcome tool except for the item of transportation.

The purpose of this study was to compare telehealth and in-person service delivery models for wheeled mobility devices in terms of functional outcomes. We hypothesized that clinically significant improvements in functional mobility measured by the Functional Mobility Assessment (FMA) will occur in individuals receiving new wheeled mobility devices via both telehealth and in-person clinic evaluations.

## METHODS

This project was designated as a quality improvement project. Approval was obtained from the Veterans Affairs Pittsburgh Healthcare System (VAPHS) Quality Improvement Committee, which provided permission to publish the results.

Prospective data were collected from telehealth visits conducted between November 2017 and July 2018 at the wheelchair seating clinic at VAPHS System. In-service training was conducted at the VAPHS Wheelchair Clinic to explain the service delivery protocol to the occupational and/or physical therapists assisting with the project. Grenier (2018) provides a comprehensive overview of the development and implementation of the service delivery protocol used for this home-based telerehabilitation assessment for wheelchair seating and mobility. The opportunity to participate in the study was presented to the Veteran when scheduling their appointment. Those who agreed to participate and fulfilled the set of criteria were screened and triaged by a treating provider for appropriateness and scheduling.

Veterans receiving telehealth services were matched to non-Veterans who received in-person assessments for wheeled mobility devices based on age within one year, gender, and primary diagnosis. Matched participants were located within the Functional Mobility Assessment (FMA) and associated Uniform Dataset (UDS) outcomes management system. This registry is a strategy developed between the University of Pittsburgh and US Rehab, which is comprised of a nationwide network of mobility equipment providers (Schmeler et al., 2019). US Rehab providers collect FMA/UDS at set multiple times following provision of a mobility device. The goal of the registry is to monitor progress, accrue large data, perform Quality Assurance, and conduct research on the effectiveness of device interventions and service delivery models.

The telehealth and in-person clinic evaluations followed the Rehabilitation Engineering and Assistive Technology Society of North America (RESNA) best-practice guidelines when choosing an appropriate device for mobility. RESNA's Wheelchair Service Provision Guide was created to show the essential steps when providing a wheelchair. It considers important factors including the current technology used, environment, support system activity, participation, body functions and structures, and the goals of the client (Arledge et al, 2011).

The FMA is a patient-reported outcome questionnaire that assesses a person's satisfaction in performing common mobility related activities of daily living such as health needs, reaching, transfers, personal care tasks, indoor mobility, outdoor mobility, and using transportation resources (Kumar et al., 2013; Paulisso et al., 2019). Each of ten items is scored on a scale of 1 (completely disagree) to 6 (completely agree) to reflect level of agreement in self-perceived satisfaction in performing the mobility-related activity of daily living. A total score is then calculated from individual items. The tool typically takes less than 5 minutes to administer and serves as an outcome measure given scores can be compared pre provision and post provision of a properly fitted device. A mean change of ≥1.85 points is considered a clinically significant change for each of the items (Schein et al., 2010; Schmeler, 2005).

The FMA was administered at two time points. Time 1 (T1) was collected upon initial assessment for a mobility device, and Time 2 (T2) was collected at least 21 days post-delivery of receiving a new mobility device. Veterans receiving telehealth assessments completed the FMA during their telehealth evaluation and then over the phone at T2 from a member of the VAPHS Wheelchair Seating team. In the clinic group, T1 scores were collected at the in-person visit with the therapist, and T2 scores were collected over the phone or by a mailed-in survey. All FMA data were collected by trained personnel.

In order to be included in the analysis, cases in both groups must have met the following criteria: FMA T1 was complete; FMA T2 was complete; and participant was seen by a credentialed Assistive Technology Professional for their clinic or telehealth evaluation.

## DATA ANALYSIS

IBM SPSS Statistics 25 was used for all analyses. Age was compared across groups using a paired samples t-test. Device used at baseline and follow up was compared across groups using Fishers Exact tests. Individual Wilcoxon signed-rank tests were used within and between groups to compare individual and total FMA item scores at T1 and T2. Change scores were calculated by taking the difference between individual item and total FMA scores at T1 and T2 for both groups, and then change scores were compared using Wilcoxon signed-rank tests. A Bonferroni correction was used for multiple comparisons where alpha level was set to 0.005 instead of 0.05 due to having 11 individual analyses (10 items and 1 total scare (0.05/11 = 0.005).

## RESULTS

A total of 43 Veterans were assessed with telehealth, but 16 lacked follow up data. Therefore, 27 Veterans were included and matched to 27 participants from the UDS. The ages reported for the two groups were 81.6 ± 8.6 (telehealth) and 79.9 ± 9.1 (clinic) years. There was not a significant difference between groups for the type of mobility devices participants use at T1 but there was a significant difference (<0.001) for the type of mobility devices prescribed between the two groups. See Table 1 for demographic information.

**Table 1:** Demographics

	Telehealth	Clinic	Z	*p*	*Statistical test*
Demographic	N = 27	N = 27			
Age (Mean ± SD)	81.63 ± 8.58	79.93 ± 9.14	0.706 (t)	0.483	Paired samples t-test
Gender (n, %)**					
Male	27 (100%)	27 (100%)			
Female	0 (0%)	0 (0%)			
Primary Diagnosis (n, %)**					
Amputation	2 (7.4%)	2 (7.4%)				
Cardiopulmonary	3 (11.1%)	3 (11.1%)			
Osteoarthritis	5 (18.5%)	5 (18.5%)			
Other Neuromuscular Conditions	8 (29.6%)	8 (29.6%)			
Parkinson Disease	1 (3.7%)	1 (3.7%)			
SCI (tetraplegia)	1 (3.7%)	1 (3.7%)			
Stroke	7 (25.9%)	7 (25.9%)			
Device at Time 1				0.342	Fishers Exact Test
No Device	1 (3.7%)	2 (7.4%)			
Transport Chair	1 (3.7%)	0 (0.0%)			
Cane/Crutches/Walker	12 (44.4%)	10 (37.0%)			
POV/Scooter	0 (0.0%)	1 (3.7%)			
K0001/K0002 MWC	0 (0.0%)	2 (7.4%)			
K0003/K0004 MWC	8 (29.6%)	1 (3.7%)			
K0009/Not Coded MWC	0 (0.0%)	1 (3.7%)			
Group 1 PWC	0 (0.0%)	1 (3.7%)			
Group 2 PWC	1 (3.7%)	6 (22.2%)			
Group 3 PWC	4 (14.8%)	3 (11.1%)			
Device at Time 2				<0.001*	Fishers Exact Test
Transport Chair	1 (3.7%)	0 (0.0%)			
K0003/K0004 MWC	0 (0.0%)	1 (3.7%)			
K0005 Ultralight MWC	10 (37.0%)	1 (3.7%)			
Tilt-in-Space MWC	2 (7.4%)	0 (0.0%)			
Group 1 PWC	1 (3.7%)	0 (0.0%)			
Group 2 PWC	0 (0.0%)	12 (0.0%)			
Group 3 PWC	12 (44.4%)	13 (48.1%)			
Group 4 PWC	1 (3.7%)	0 (0.0%)			

* statistically significant at 0.005 level

** p value not reported due to matching

SD = standard deviation; SCI = spinal cord injury; POV = power operated vehicle; MWC = manual wheelchair; and PWC = power wheelchair

Table 2 shows results of comparing baseline FMA scores between the two groups. Six participants marked ‘Does Not Apply' for one individual item, and two marked ‘Does Not Apply' for two items (1.8% responses). No statistically significant differences were found in FMA scores at T1.

**Table 2:** Time 1 FMA Mean Scores

	Telehealth	Clinic	*p*
FMA Item	N = 27	N = 27	
Daily Routine	3.56 (1.91)	2.44 (1.76)	0.013
Comfort	3.26 (1.72)	2.46 (1.96)	0.154
Health	3.33 (1.80)	2.69 (1.87)	0.304
Operate	3.67 (1.82)	2.81 (1.92)	0.119
Reach	2.89 (1.85)	2.92 (1.89)	0.988
Transfers	3.93 (1.62)	3.46 (1.79)	0.351
Personal Care	4.11 (1.67)	2.88 (1.64)	0.009
Indoor Mobility	4.44 (1.40)	3.11 (1.78)	0.013
Outdoor Mobility	2.00 (1.54)	2.44 (1.85)	0.366
Transportation	3.30 (1.98)	3.21 (1.69)	0.687
Total	34.48 (12.91)	27.33 (15.59)	0.141

FMA = Functional Mobility Assessment

* statistically significant at 0.005 using Wilcoxon signed rank tests and Bonferroni correction

Table 3 shows results of comparing T2 FMA scores between the two groups. One participant marked ‘Does Not Apply' for one individual FMA item (0.2% of responses). The FMA score for transfers was significantly higher in the telehealth group than the clinic group, but no other FMA scores were significantly different.

**Table 3:** Time 2 FMA Mean Scores

	Telehealth FMA	Clinic FMA	*p*
FMA Item	N = 27	N = 27	
Daily Routine	5.59 (0.69)	4.89 (1.85)	0.126
Comfort	5.52 (1.09)	4.93 (1.82)	0.154
Health	5.81 (0.62)	5.15 (1.79)	0.107
Operate	5.70 (0.67)	5.00 (1.75)	0.129
Reach	5.56 (0.97)	4.70 (1.81)	0.063
Transfers	5.93 (0.27)	4.93 (1.66)	0.002*
Personal Care	5.74 (0.86)	4.89 (1.93)	0.055
Indoor Mobility	5.78 (0.42)	5.44 (1.37)	0.458
Outdoor Mobility	5.52 (0.85)	4.81 (1.90)	0.108
Transportation	5.56 (0.97)	4.31 (2.15)	0.014
Total	56.70 (4.72)	48.89 (14.70)	0.025

* statistically significant at 0.005 using Wilcoxon signed-rank tests and Bonferroni correction

FMA = Functional Mobility Assessment

**Figure 1:**
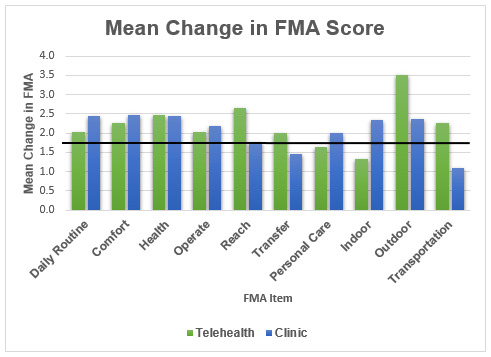
Mean Change in FMA Scores

All mean individual item and total FMA scores in both groups increased from T1 to T2. Figure 1 shows the mean change in each of the individual FMA item scores with a solid horizontal black line at the clinically significant threshold change of 1.85. Within the telehealth group, all changes in individual item and total FMA scores were statistically significant, with changes in 8 of 10 items meeting threshold for clinical significance (change ≥1.85 points) (personal care and indoor mobility did not reach threshold for clinically significant improvement). Within the clinic group, changes in 7 of 10 individual items and total FMA scores were statistically significant, and these same seven items met threshold for clinical significance (reach, transfers, and transportation). Table 4 reports p values for comparison of individual item and total FMA scores within each group. Change scores for individual item and total FMA scores did not differ significantly between the two groups.

**Table 4:** Significance levels of FMA Scores at Time 1 Compared to Time 2 within Both Groups

	Telehealth	Clinic
FMA Item	*p*	*p*
Daily Routine	<0.001*	<0.001*
Comfort	<0.001*	0.001*
Health	<0.001*	0.001*
Operate	<0.001*	0.001*
Reach	<0.001*	0.005
Transfers	<0.001*	0.008
Personal Care	0.001*	0.003*
Indoor Mobility	<0.001*	<0.001*
Outdoor Mobility	<0.001*	0.001*
Transportation	<0.001*	0.074
Total	<0.001*	<0.001*

* statistically significant at 0.005 using Wilcoxon signed-rank tests and Bonferroni correction

FMA = Functional Mobility Assessment

## DISCUSSION

For people with mobility impairments, access to care and to practitioners with special training in wheeled mobility and seating is difficult and cumbersome (Cooper et al., 1996; Hoenig et al., 2005). Telehealth is not intended to supplant existing traditional wheeled mobility and seating assessments, but rather to provide an alternative method of delivering services. Similar to Cooper et al. (2002) when prescribing a particular device under telehealth, this study accounted for the wheelchair users' health condition (i.e., diagnosis) and complexity as it relates to their FMA score on specific functional items. In addition, similar results to Dallolio et al. (2008) where authors noted some improvements in grooming, dressing and transfer tasks after discharge, this study found a clinical significance in specific functional tasks such as daily routine, comfort, health, operate, personal care, and outdoor mobility via telehealth or traditional in-person after a new wheeled mobility device was prescribed.

This study demonstrated increases in all FMA items and total score, regardless of whether the visit was conducted in clinic or via telehealth. Similar to Schein et al. (2010) which found no significant differences in Functioning Everyday with a Wheelchair outcome item scores at pre or posttest (except for transportation), the authors found no significant differences at T1 or T2 except for transfer measured by the FMA. The FMA is a derivative of the FEW developed by researchers at the University of Pittsburgh. A likely reason why change scores for individual items and total FMA scores did not differ significantly between the two groups were that both groups at T1 and T2 were already using high-end customized manual and power wheelchairs. In the telehealth group, increases in indoor mobility and personal care items were statistically significant but likely did not reach threshold for a clinically significant change because they were already relatively high at baseline. In the clinic group, the changes in the reach and transportation domains likely did not reach threshold for clinically or statistically significant change because the majority of individuals received power wheelchairs with various seat functions but not specifically seat elevation, which may not have facilitated reaching and may have been more difficult to transport. This could be due to the absence of funding for seat elevators and vehicle lifts, both of which are out-of-pocket expenses to participants within the clinic group which followed Medicare insurance policies and regulations. Therefore, participants would not see as great improvements in functional tasks such as transfers, reach, and transportation. Compared to the telehealth group where Veterans Administration (VA) insurance policies and regulations differ in terms of what accessories (e.g., seat elevator and vehicle lifts) are covered benefits. In addition, the higher change in improvement on outdoor mobility for the telehealth group can likely be attributed again to the difference in funding regulations between the VA and Medicare insurance. Through Medicare and some other insurance policies (used in the clinic setting), these funding sources are mostly concerned with mobility use inside the home only. The FMA domain for transfers did not improve to a clinically or statistically significant level in the clinic group but did improve to these thresholds in the telehealth group. The transfer score was also significantly higher at T2 in the telehealth group compared to the clinic group, possibly suggesting that telehealth visits confer some advantages in being able to assess and address transfer issues in the home.

## STUDY LIMITATIONS

Several limitations deserve discussion. First, even though the groups were matched on diagnosis, age, and gender, they were not perfectly matched. One group included Veterans receiving devices in a VA system, while the other included civilians receiving devices in a non-VA system. Specifically, funding policies for mobility devices were different between the two groups. Although the groups started out with a similar distribution of devices, the distribution of types of devices that they received were different. Therefore, the improvements seen in both groups may not necessarily be entirely due to the method of service delivery. However, all individuals were assessed by trained certified Assistive Technology Professionals using best practice protocols, and a majority of individuals in both groups received high quality devices, such as ultralight manual wheelchairs, or Group 2 to 4 power wheelchairs, suggesting the quality of evaluation or technology is less likely to have affected outcomes. A second limitation is that the FMA was administered across the two groups in different settings. However, strict protocols as described by Grenier (2018) and Schmeler (2019) were followed. Third, our methods increased the likelihood of a Type II error, but the consistent trends seen between and within groups provide some confidence that similar trends would be seen with larger samples. Lastly a ceiling effect may have limited our ability to detect changes in some FMA scores.

## CONCLUSION

The study compared telehealth and in-person service delivery models for wheeled mobility devices in terms of functional outcomes measured by the FMA. Telehealth is not intended to supplant existing traditional wheeled mobility and seating assessments but rather to provide an alternative method of delivering services. Using telehealth in the home setting allowed therapists to not only evaluate the client in their natural environment, but to also determine the accessibility of that environment. The majority of FMA items displayed clinically significant increases after provision of a mobility device via telehealth and in-person clinic evaluation. Specifically, a larger and clinically significant change in transfer score was seen in the telehealth group, suggesting telehealth visits may confer some advantages in being able to assess and address transfer issues in the home.

## Notes

This project was funded as a FY17 MyVA Access Improvement Project through The Office of the Under Secretary for Health (USH) and the Office of Rural Health (URH) in collaboration with The Office of Veterans Access to Care (OVAC).
